# The Focal Adhesion-Localized CdGAP Regulates Matrix Rigidity Sensing and Durotaxis

**DOI:** 10.1371/journal.pone.0091815

**Published:** 2014-03-14

**Authors:** Duncan B. Wormer, Kevin A. Davis, James H. Henderson, Christopher E. Turner

**Affiliations:** 1 Department of Cell and Developmental Biology, State University of New York Upstate Medical University, Syracuse, New York, United States of America; 2 Syracuse Biomaterials Institute, Syracuse University, Syracuse, New York, United States of America; Seoul National University, Republic of Korea

## Abstract

Motile cells are capable of sensing the stiffness of the surrounding extracellular matrix through integrin-mediated focal adhesions and migrate towards regions of higher rigidity in a process known as durotaxis. Durotaxis plays an important role in normal development and disease progression, including tumor invasion and metastasis. However, the signaling mechanisms underlying focal adhesion-mediated rigidity sensing and durotaxis are poorly understood. Utilizing matrix-coated polydimethylsiloxane gels to manipulate substrate compliance, we show that cdGAP, an adhesion-localized Rac1 and Cdc42 specific GTPase activating protein, is necessary for U2OS osteosarcoma cells to coordinate cell shape changes and migration as a function of extracellular matrix stiffness. CdGAP regulated rigidity-dependent motility by controlling membrane protrusion and adhesion dynamics, as well as by modulating Rac1 activity. CdGAP was also found to be necessary for U2OS cell durotaxis. Taken together, these data identify cdGAP as an important component of an integrin-mediated signaling pathway that senses and responds to mechanical cues in the extracellular matrix in order to coordinate directed cell motility.

## Introduction

Cells derive signals from interaction with the surrounding extracellular matrix (ECM) to regulate crucial functions including cell growth, differentiation and motility [1]. Integrin binding to glycoproteins present in the ECM, such as collagen and fibronectin, stimulates cell motility and promotes the formation of focal adhesions (FAs) in part by signaling to the intracellular Rho family of GTPases, including Rac1, RhoA, and Cdc42 [2]. These molecular switches are activated by guanine nucleotide exchange factors (GEFs) and inactivated by GTPase activating proteins (GAPs) during cell migration to coordinate signaling to the cellular migration machinery, including the regulation of FA dynamics and the remodeling of the actomyosin cytoskeleton through activation of downstream Rho family effectors such as PAK, Arp2/3, and non-muscle myosin II isoforms [3]–[7].

In addition to its chemical composition, recent studies have shown that the mechanical properties of the ECM also influence integrin signaling to promote directed cell migration [8]–[10]. Specifically, cell motility rates are enhanced by increased matrix rigidity and cell migration is directed towards more rigid substrates in a process known as durotaxis [8]–[10]. Artificially changing ECM compliance or exerting experimentally derived force on integrins can regulate the Rho family GTPases RhoA and Rac1, suggesting that ECM rigidity activates integrin signaling to control the Rho family of GTPases [11]–[14]. However, the function and activity of Rho GTPases during mechanically directed cell migration remains unclear and furthermore, the specific GEFs and GAPs that modulate their spatial and temporal activity to promote durotaxis have not been identified.

CdGAP is a Rac1 and Cdc42 specific GAP that localizes to FAs formed on rigid surfaces to regulate cell migration, FA size, and FA dynamics in an integrin- dependent manner [15], [16]. CdGAP also regulates cell migration within more compliant 3D cell-derived extracellular matrices [15]. Mutations in cdGAP are causative for defects in vasculogenesis, heart formation, skin wound closure and limb formation that comprise the syndrome known as Adams-Oliver disease, a disorder which may be the result of altered rigidity sensing or dysregulated stem cell migration and differentiation [9], [17]. CdGAP also plays a role in cancer, where changes in ECM stiffness and rigidity sensing promote metastasis [15], [18]–[20].

Using Polydimethylsiloxane (PDMS)-based gels of different rigidity, we determined that cdGAP is necessary for optimal rigidity sensing, driving changes to the migration machinery as a function of ECM compliance and thereby enhancing rigidity-dependent cell migration and durotaxis.

## Results

### CdGAP Regulates Cell Morphology and Motility in an ECM Rigidity-Dependent Manner

**Figure 1:**
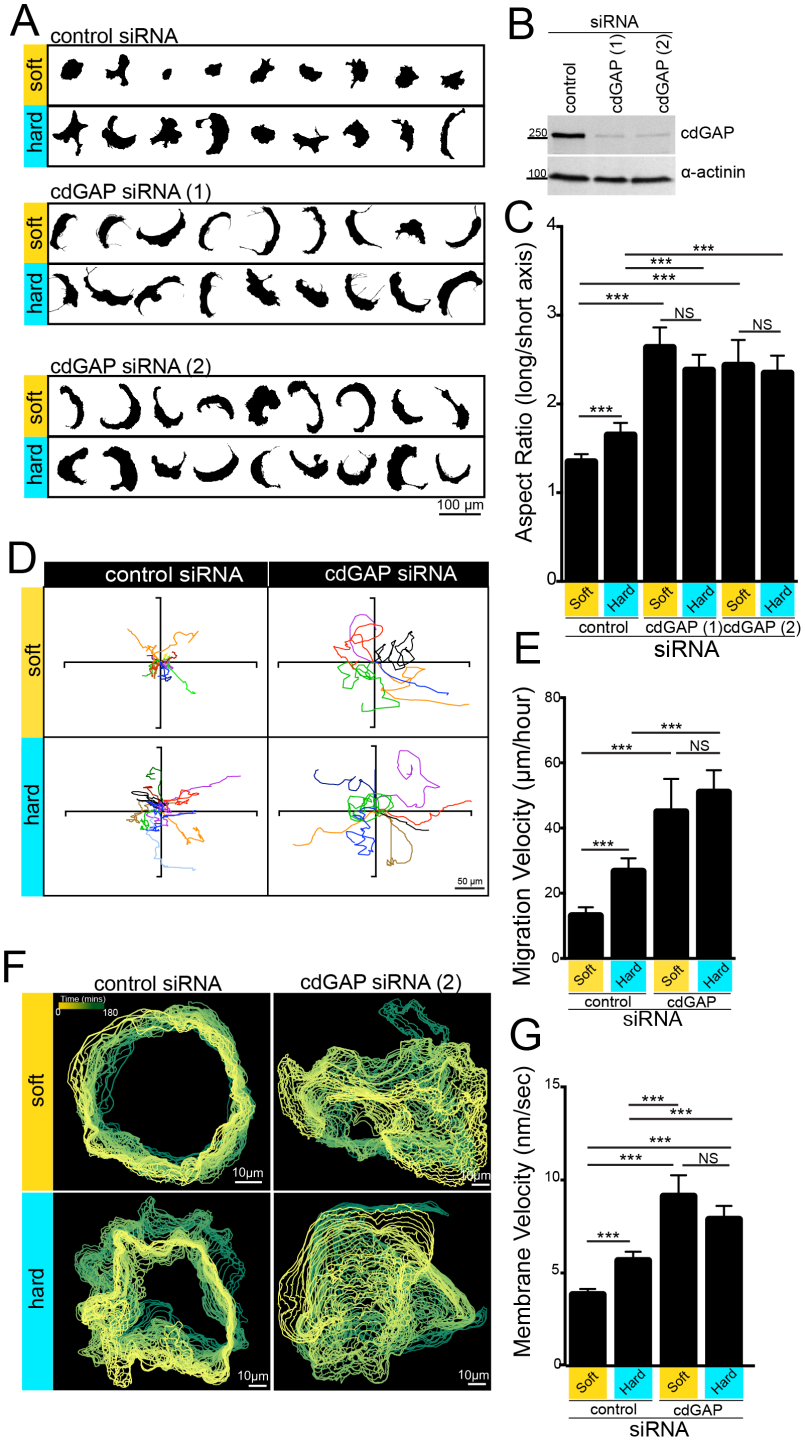
CdGAP Regulates Cell Morphology, Motility, and Membrane Dynamics in a Matrix Rigidity-Dependent Manner. (**A**) U2OS cells treated with control and two independent cdGAP siRNAs were plated on soft and hard PDMS substrates coated with fibronectin. Cells were stained for F-actin and masks created of the thresholded actin images. (**B**) Transfection of two independent cdGAP siRNAs efficiently suppressed cdGAP protein expression in U2OS cells. (**C**) Control siRNA-treated cells increased their aspect ratio (long:short axis of the cell) significantly in response to hard substrates, whereas cdGAP siRNA-treated cells maintained an exaggerated crescent morphology with a higher aspect ratio than controls and did not change their aspect ratio as a function of matrix rigidity. (**D**) U2OS cells were transfected with control or cdGAP siRNA and plated onto soft or hard PDMS coated coverslips and individual cells tracked over the course of 16 hour movies to determine cell migration velocity. (**E**) Control siRNA-treated cells migrated at a higher velocity on hard substrates, whereas the migration of cdGAP siRNA-treated cells was substantially elevated above that of control cells on both soft and hard substrates. (**F**) Control siRNA-treated and cdGAP-depleted cells were imaged at high magnification and montages of membrane dynamics were compiled over 20 minute periods using the Quimp11 plugin for ImageJ. (**G**) Overall membrane protrusion and retraction velocity for control and cdGAP siRNA-treated cells, demonstrating that control siRNA-treated cell membranes are more dynamic on a hard substrate, whereas cdGAP siRNA caused cells to have equally dynamic membranes on soft and hard substrates and rapid membrane movement as compared to control cells. For spread area, aspect ratio, and cell migration analysis, a total of 15–30 cells from three independent experiments were analyzed. For Quimp11 analysis averages represent 3–6 cells from three independent experiments over a 20 minute period.

U2OS osteosarcoma cells respond to integrin-ECM interaction on rigid glass substrates by spreading and then becoming highly motile, adopting an atypical crescent shaped morphology [15], [16], [21]. Thus, crescent-shaped U2OS cells have a long axis as measured from side to side and shorter minor axis as measured from the leading edge to the rear of the cell, giving them a distinctive high aspect ratio (long:short axis of the cell) as compared to the wedge shape typical of migrating fibroblasts. Perturbing cdGAP expression levels via overexpression or siRNA has previously been shown to regulate cell spreading and the ability of U2OS cells to attain a crescent phenotype in response to integrin-ECM engagement on traditional rigid glass or tissue culture substrates [15], [16]. To determine if cdGAP also controls U2OS morphology as a function of ECM rigidity, we generated soft PDMS substrates of 1 kPa and hard 1 MPa that mimicked the approximate elastic modulus in-vivo of interstitial connective tissue and bone, respectively [22]. On soft substrates, control small interfering RNA (siRNA)-treated U2OS cells had a reduced spread area (Figure S1A) and a rounded morphology with a low aspect ratio (Figure 1A,C) whereas on hard substrates cells were well spread with a crescent morphology (Figure 1A,C). In contrast, cdGAP siRNA-treated cells (Figure 1B) were unable to detect and respond to the compliance of the soft ECM and exhibited an equivalent spread area (Figure S1A) and also demonstrated an exaggerated crescent morphology (high aspect ratio) on both soft and hard substrates (Figure 1A,C).

We next determined if cdGAP also regulated cell motility as a function of matrix rigidity. The majority of control U2OS cells on soft substrates either remained rounded for the duration of the migration analysis or transiently established a leading edge and migrated at a low speed (Figure 1D,E and Movie S1), whereas on hard substrates, they transitioned to, and maintained, a crescent morphology and migrated at significantly increased rates (Figure 1D,E and Movie S2). Conversely, cdGAP RNAi-treated cells were unresponsive to rigidity changes and migrated at an accelerated rate on either compliant or rigid substrates with an exaggerated crescent morphology that changed rapidly over time (Figure 1D,E and Movies S3,S4).

The rate at which cells migrate correlates with their ability to extend, stabilize, and retract the plasma membrane [6], [23], [24]. Furthermore, regulation of membrane extension and retraction rates has been associated with cells that are capable of ECM rigidity sensing, such as fibroblasts and stem cells [22], [25]–[27]. Control cells plated on soft substrates slowly extended and retracted their membrane (Figure 1F,G) compared to control cells on hard substrates (Figure 1F,G). In contrast, cdGAP RNAi cells were unaffected by ECM rigidity and extended and retracted their membrane on both compliant and rigid surfaces at significantly faster rates than control cells (Figure 1F,G).

Together, these data indicate that cdGAP plays a central role in suppressing the transition of U2OS cells to a motile phenotype on soft substrates of 1 kPa, resulting in inhibition of cell migration rates, whereas cells depleted of cdGAP are unresponsive to changes in matrix rigidity between 1 kPa and 1 MPa. Furthermore, the accelerated rates of membrane protrusion and retraction in cdGAP-depleted cells may contribute to a reduced capacity of these cells to sense matrix rigidity via differences in integrin-ECM signaling.

### CdGAP Regulates FA Organization and Dynamics in Response to ECM Matrix Rigidity

Exposure of cells to rigid ECM increases non-muscle myosin IIA activity to promote cytoskeletal contractility and results in an overall increase in FA size [56]. However, the effects of mechanical signals originating from the ECM on FA dynamics are unclear, as they have been reported to both increase and decrease on compliant ECM [27]–[30]. Nevertheless, the FA lifetime and rates of FA assembly and disassembly are crucial determinants of cell migration velocity [30]–[35]. Manipulation of cdGAP expression was previously shown to control FA size and FA dynamics in response to integrin-ECM engagement on glass substrates, so we determined if cdGAP could also regulate FAs as a function of matrix rigidity [15].

**Figure 2:**
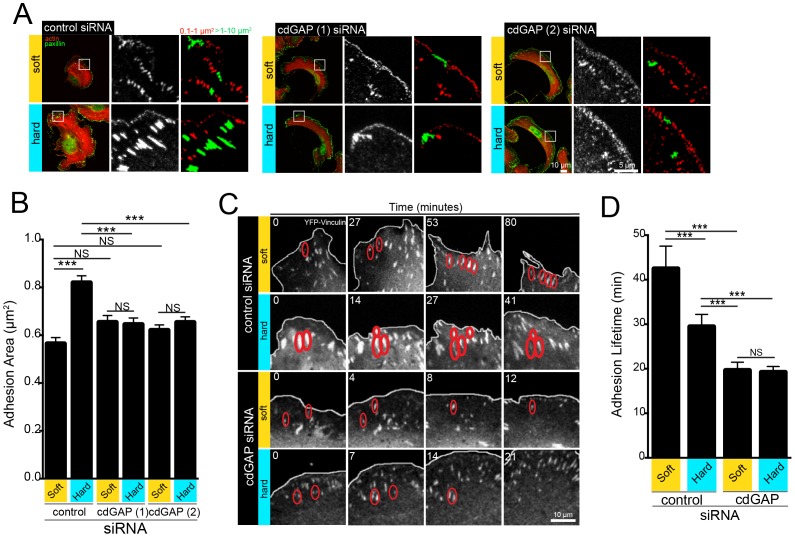
CdGAP Controls FA Size and Regulates FA Dynamics in a Rigidity-Dependent Manner. (**A**) Immunofluorescence of focal adhesions on soft versus hard PDMS. Merged images are of actin (red), and paxillin (green). Insets show pseudo-colored masks of different sized focal adhesions, with adhesions from 0.1–1 μm^2^ (Red), and adhesions >1–10 μm^2^ (Green). (**B**) Quantification of average adhesion area (μm^2^) in control versus cdGAP siRNA-treated cells plated on substrates of varying rigidity. Control cells expressing cdGAP undergo a significant increase in average adhesion size when plated onto rigid PDMS, whereas cdGAP-depleted cells maintain mostly small, peripheral adhesions on both soft and hard PDMS. (**C**) Cells expressing vinculin-YFP and treated with control or cdGAP siRNA were imaged on soft versus hard PDMS matrices to quantify their adhesion dynamics and montages of images at the indicated timepoints were generated from live-cell movies. (**D**) Adhesion lifetime was shortened dramatically in cdGAP-depleted cells on both soft and hard matrices, whereas control cells exhibited a significant decrease in adhesion lifetime when comparing cells plated on soft versus hard matrix. P-values represent student's t-test on the pooled data from two experiments using vinculin-YFP as an adhesion marker and one experiment using zyxin-GFP. Adhesion size analysis was performed on ∼1,000 total FAs from 20–30 cells from three independent experiments. For lifetime analysis, ∼100 total FAs from 3–6 cells were evaluated per experimental condition.

Control RNAi-treated cells plated on soft substrates had small adhesions (Figure 2A,B), and relatively long FA lifetimes (Figure 2C,D and Movie S5), whereas FAs of cells on hard ECM increased in size (Figure 2A,B) and turned over more quickly, resulting in shortened FA lifetimes (Figure 2A,B and Movie S6). In contrast, cdGAP RNAi-treated cells had small leading edge adhesions that failed to increase in size upon exposure to more rigid substrates (Figure 2A,B) and rapidly turned over (Figure 2C,D and Movies S7,S8). Furthermore, adhesion lifetimes in cdGAP-depleted cells on either soft or hard substrates were significantly decreased when compared to control cells (Figure 2C,D). Taken together, these data indicate that cdGAP functions to regulate FA organization and dynamics in response to matrix rigidity, enhancing FA size and controlling FA stability in a rigidity-dependent fashion.

### CdGAP Regulates Rac1 Activity in an ECM Rigidity-Dependent Fashion

**Figure 3:**
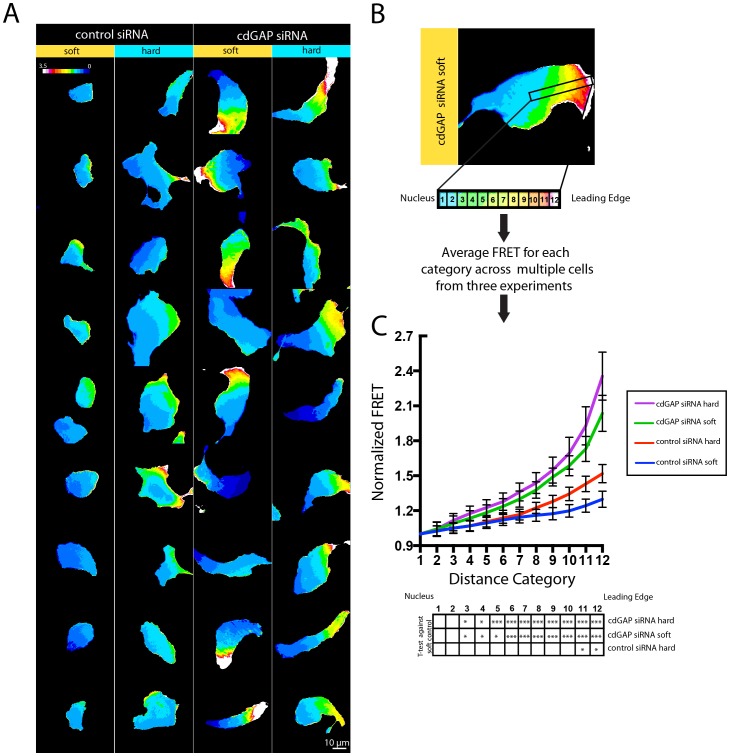
CdGAP Inhibits the Spatial Activity of Rac1 in a Rigidity-Dependent Manner. (**A**) The Raichu Rac1 FRET biosensor was transfected into control and cdGAP siRNA-treated cells and cells were plated onto either soft or hard PDMS substrates. Fluorescence images of live cells were acquired and the ratio of CFP/YFP images was taken for each cell to generate a color enhanced image. Pseudo-colored scales were set with the same range for all images. (**B**) Line profiles 20 μm wide were generated from the leading edge to the nucleus and the relative FRET efficiency was calculated along each line profile. To normalize for differences in cell size, groups of FRET values along the linescan were binned into twelve distance categories and averaged from three independent experiments to produce the FRET gradients. (**C**) Control cells demonstrated graded increases in Rac1 activity from the nucleus to the leading edge on rigid substrates, whereas cdGAP siRNA-treated cells consistently displayed a steeper gradient of Rac1 activity at the very leading edge of migrating cells that was unchanged when cells were plated onto either soft or rigid substrates. The statistical analysis in the table in Figure 3 was performed by determining a student's t-test relative to the control cell values for each distance category on soft PDMS. Distance categories 3–12 were all significantly higher for cdGAP siRNA-treated cells on soft and hard matrices when compared to control cells on soft matrices. Control cells plated on hard matrices had significantly enhanced FRET in categories 10–12 when compared to control cells plated onto soft PDMS. A separate statistical t-test analysis comparing cdGAP-depleted cells plated on soft versus rigid substrates showed that there was no significant difference for any distance category. At least fifteen cells from three independent experiments were analyzed for each average FRET gradient shown in (C).

Rac1 and the Rac1 isoform Rac1b, as well as the Rac1 homologue Ced-10 (*D. Melanogaster*), are activated in-vitro and in-vivo in response to force or as a function of matrix compliance [12]–[14], [36]. We previously showed that integrin-ECM interactions stimulated cdGAP's GAP activity towards Rac1, so we determined whether ECM rigidity could also influence cdGAP's ability to regulate Rac1 activity [16]. Comparison of FRET signals from the Raichu-Rac1 biosensor in control RNAi-treated cells on soft and rigid substrates revealed a significant increase in the gradient of active Rac1 in cells at the leading edge on rigid substrates (Figure 3A–C). In contrast, cdGAP knockdown cells were again unresponsive to matrix rigidity, with no significant difference in the Rac1 activity gradient in cells plated on soft versus hard substrates (Figure 3A–C). In addition, cdGAP RNAi-treated cells also exhibited dramatically enhanced gradients of Rac1 activation when compared to control siRNA-treated cells on either soft or hard surfaces (Figure 3A–C). Given that activated Rac1 has been shown to enhance formation of a crescent morphology in U2OS cells, and decrease FA lifetimes independently of Cdc42, these data indicate that cdGAP inhibits localized Rac1 activity at the leading edge as a function of matrix rigidity. In turn, inhibition of Rac1 potentially suppresses both lamellipodia formation and FA dynamics, resulting in rigidity-dependent cell migration [15], [33], [35], [37], [38].

### CdGAP is necessary for Durotaxis

**Figure 4:**
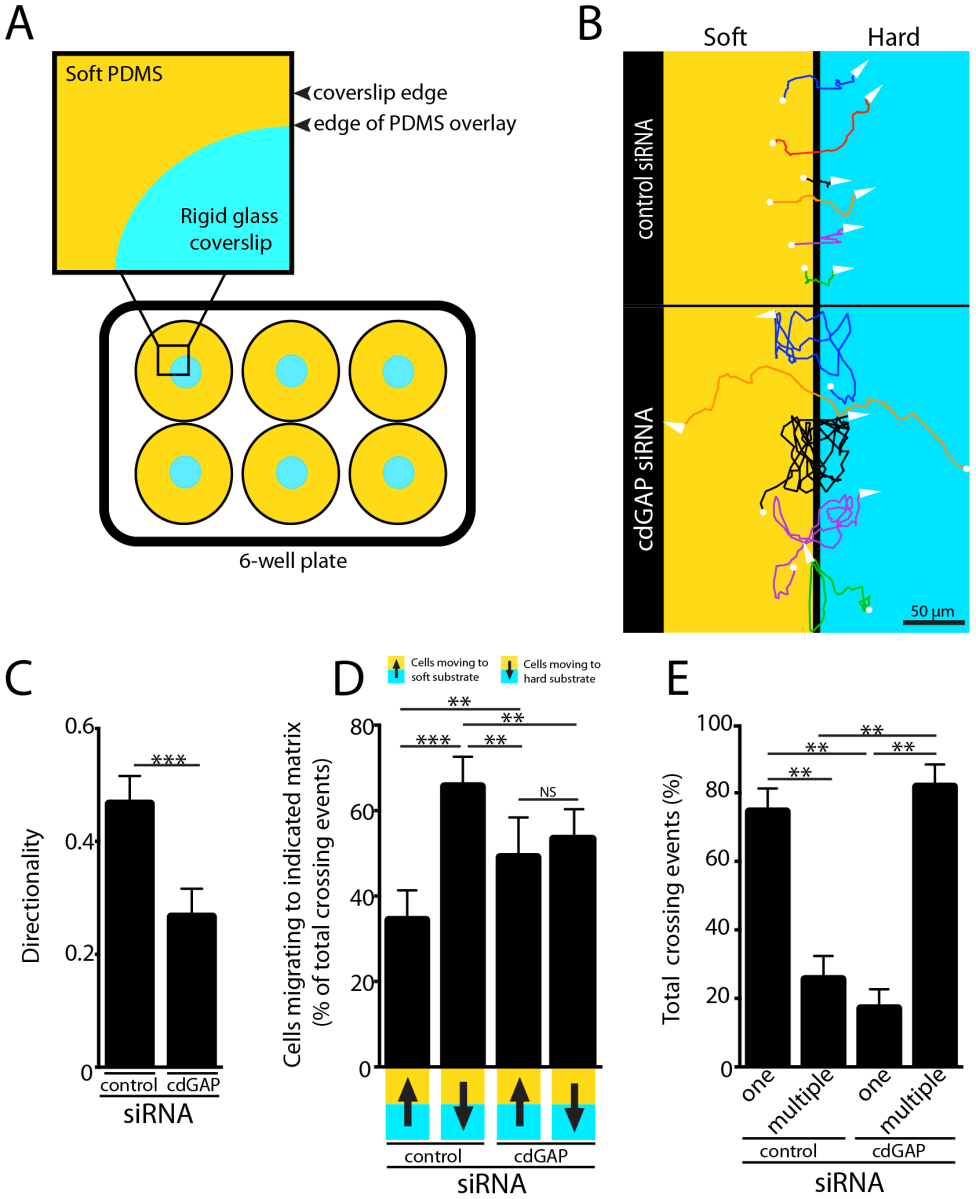
CdGAP is Necessary for Durotaxis. (**A**) Cell durotaxis was measured across a glass-soft PDMS interface, (see materials and methods) (**B**) Representative tracks of control and cdGAP siRNA-treated cells plated into durotaxis chambers tracked over the course of 16 hours. (**C**) CdGAP siRNA-treated cells that crossed the rigidity interface moved with less directionality than control cells crossing the rigidity boundary. (**D**) Control siRNA-treated cells crossed onto the glass coverslip preferentially, where they typically remained for the duration of each experiment. In contrast, cdGAP siRNA treatment resulted in equivalent numbers of cells crossing from soft to hard and hard to soft, diminishing the ability of migratory cells to differentiate between soft and rigid substrates. (**E**) Control siRNA-treated cells typically crossed the rigidity interface only once, whereas cdGAP siRNA treatment promoted multiple crossings of migrating cells in either direction. Crossing data represent a minimum of 150 total cell crossings for each experimental condition from three independent experiments.

Durotaxis, or the preferential movement of cells from a compliant to more rigid environment, requires coordinated changes to the cell migration machinery as a function of matrix rigidity, involving the force-dependent increase in size of FAs, as well as regulation of the contractility of the actin cytoskeleton during lamellipodia formation [8], [39], [40]. Our data indicated that these components of the cell migration machinery were unresponsive to matrix rigidity in cdGAP RNAi-treated cells, so we determined if cdGAP was necessary for durotaxis. Control RNAi-treated cells plated into a durotaxis chamber with a soft:hard rigidity interface (Figure 4A, see Materials and Methods for details) preferentially migrated towards the more rigid glass surface (Figure 4B–D, Movie S9). Conversely, cells depleted of cdGAP migrated with significantly reduced directionality (Figure 4B,C) and without preference for soft or hard ECM (Figure 4D, Movie S10). In addition to losing their preference for more rigid substrates, cdGAP siRNA-treated cells crossed the rigidity interface significantly more frequently than control cells (Figure 4E, Movie S10), a further indication that these cells migrated with diminished ability to actively sense and respond to changes in ECM rigidity. Importantly, the differences in cell crossing rates we observed were not the result of a bias in the number of cells adherent to either the soft or hard side of the rigidity interface (Figure S1B). Together, these data indicate that cdGAP expression is essential for individual cells to actively differentiate between ECM of varying rigidity and to demonstrate efficient durotaxis.

## Discussion

The FA protein cdGAP was previously identified as a suppressor of cell spreading and crescent morphology in U2OS cells in response to the engagement and activation of integrins by ECM proteins absorbed onto traditional rigid glass or plastic cell culture substrates [16]. Herein, we show that cdGAP not only responds to integrin engagement of the ECM, but that it is essential for matrix rigidity sensing of ECM substrates between 1 kPa and 1 MPa. Specifically, we found that cdGAP is a suppressor of cell spreading and the transition to a motile phenotype in cells plated on soft substrates.

CdGAP is recruited to FAs via an interaction with actopaxin (alpha-parvin), which is part of a complex including the integrin-linked kinase (ILK) [16], [41]–[43]. Importantly, the interaction between cdGAP and actopaxin is required for cdGAP's inhibitory effects on crescent formation in U2OS cells [16]. Moreover, depending on its phosphorylation state, actopaxin can either promote or inhibit crescent formation and cell migration in U2OS cells [16], [38], [44]. Taken together with recent studies showing that ILK and actopaxin are recruited into FAs in an integrin-specific and rigidity-dependent manner in order to promote rigidity sensing on fibronectin, this suggests that the ILK-actopaxin complex and cdGAP may form a mechanically sensitive signaling axis that controls U2OS cell morphology and motility in response to ECM compliance [45], [46].

Interestingly, cdGAP inhibited crescent formation and cell motility to a greater extent on soft substrates, suggesting that increased rigidity may perturb the activity or dynamics of cdGAP and/or ILK-actopaxin signaling. The mitogen-activated kinase ERK is activated by increased ECM rigidity, and active ERK has been shown to directly bind to and phosphorylate cdGAP on multiple residues including threonine 776, resulting in the inhibition of cdGAP's GAP activity towards both Rac1 and Cdc42 [19], [45], [47]. ERK also phosphorylates the actopaxin amino-terminus, resulting in the transition of U2OS cells to a crescent phenotype through PIX-dependent activation of Rac1 and PAK [44], [48]. Thus, increased ERK kinase activity on more rigid substrates could potentially reduce cdGAP's inhibition of Rac1 or Cdc42 and enhance the phosphorylation of actopaxin's amino-terminus to promote crescent formation and cell migration.

Using Raichu FRET analysis as a reporter for Rac1 activity, we found that cdGAP plays an essential role in regulating Rac1 activity at the leading edge and is to our knowledge the first FA localized Rac1 GAP that has been shown to be regulated by matrix rigidity. Interestingly, knockdown of other Rac1 GAPs that respond to integrin-ECM engagement, including SrGAP1 and Sh3BP1, broadened the spatial gradient of Rac1 activity at the leading edge and resulted in enhanced protrusion and migration [49], [50]. Furthermore, spatially restricted activation of Rac1 at the membrane or regulation of the gradient of Rac1 activity at the leading edge results in the formation of new lamellipodia and alters cell migration rates [51]–[53]. Thus, by suppressing Rac1 activity at the leading edge in a rigidity-dependent manner, cdGAP may determine both the capacity of U2OS cells to transition into a crescent morphology and control membrane protrusive activity to regulate cell migration. Alternatively, cdGAP has also been shown to regulate the activity of Cdc42, which enhances lamellipodia and filopodia formation as well as cell motility, so we cannot exclude a role for Cdc42 in rigidity-dependent U2OS cell crescent formation and motility.

Rac1 and Cdc42 activation at the leading edge drives the initial formation of small adhesions in an actin polymerization-dependent mechanism [23], [54]. RhoA is also localized to the leading edge and is activated spatiotemporally ahead of Rac1, but in contrast promotes the formation of contractile stress fibers resulting in the formation of large, long-lived FAs [32], [30], [56], [57]. CdGAP knockdown led to the formation of small FAs independent of matrix rigidity, suggesting that FAs in cdGAP RNAi-treated cells may have failed to mature due to decreased RhoA activity and/or the failure to activate downstream RhoA effectors such as ROCK or non-muscle myosin IIA. Alternatively, small FAs also result from the destabilizing effects of activated Rac1, which can drive rapid FA assembly and disassembly rates leading to the shortened FA lifetimes that we observed in cdGAP RNAi-treated cells plated on soft and rigid ECM. Conversely, cdGAP's suppression of Rac1 activity alone would slow FA assembly and disassembly rates and thereby could explain the lengthened FA lifetimes we observed on soft ECM in control RNAi-treated U2OS cells.

Cell motility and durotaxis are driven by traction forces exerted through FAs. Recent studies showed that a single cell straddling both soft and rigid substrates in a durotaxis assay generates asymmetric traction forces; with FAs on rigid ECM exerting high force and FAs over soft ECM exerting low forces. This in turn creates an asymmetry in traction force that leads to directed cell movement in the direction of the more rigid substrate [40], [58], [59]. Individual U2OS cells plated on soft and rigid substrates contain small and large adhesions, presumably at different levels of maturation, that in turn exhibit a broad range of traction forces [15], [29]. The data presented herein show that cdGAP RNAi-treated cells have an abundance of small adhesions, which in other studies have been shown to exert a narrower range of traction forces [29], [60]. It is interesting to speculate that cdGAP siRNA-treated cells are incapable of generating asymmetric traction forces on soft and rigid substrates due to their inability to alter FA size in response to ECM compliance and leading to rigidity-independent cell migration.

Asymmetry in traction force during durotaxis has also been observed at the level of individual FAs. The generation of traction forces within FAs is essential for durotaxis, and the peak traction force must oscillate to the anterior of individual FAs in order for cells to durotax [61], [62]. The rapid and cyclical nature of these force fluctuations within individual FAs suggests that they might be controlled by Rho GTPase signaling. Indeed, cyclical fluctuations in Rho GTPase signaling have been observed at the leading edge of randomly migrating cells and polarized cells during wound closure [55], [63]–[64]. Asymmetric force fluctuations within FAs requires paxillin phosphorylation at both tyrosine 31 and 118, which in turn can enhance the binding of Crk and recruitment of Dock 180, to potentially activate Rac1 [65]–[67]. The paxillin LD1 motif, which is just amino-terminal to Y31, recruits complexes containing ILK, actopaxin, and cdGAP to FAs [16], [41]–[43]. Thus, cdGAP may be an important component of this scaffold and signaling complex that controls the cycling of Rac1 through its GTP and GDP bound states and thereby regulates force fluctuations in FAs, rigidity sensing, and durotaxis.

To summarize, we have identified the Rac1- and Cdc42-specific GAP, cdGAP as a key mediator of FA based mechanosensing of the ECM and as an important regulator of durotaxis in U2OS osteosarcoma cells. It will be important in future studies to understand how cdGAP's function in rigidity-dependent adhesion maturation and durotaxis may be influenced by regulation of its GAP activity or through altered protein-protein interactions with actopaxin or as yet undescribed binding partners in FAs. Recently identified mutations to cdGAP in cancer patients (Cancer Genome ATLAS Project), as well as those that have been reported in Adams-Oliver Syndrome, may also provide an interesting basis for further understanding the mechanisms underlying cdGAP's regulation of mechanosensing and how specific defects in the cellular response to rigidity can lead to disease [17].

## Materials and Methods

### Cell Culture, Antibodies and Transfection

U2OS osteosarcoma cells were originally derived from a moderately differentiated tibial osteosarcoma, and for this study were obtained from the American Tissue Culture Collection (ATCC, Manassas, VA). U2OS cells were maintained in Dulbecco's Minimum Essential Media (DMEM) supplemented with 2 mM L-glutamine, 10% FBS (v/v), 10 I.U./ml penicillin, 10 μg/ml streptomycin, and 1 mM sodium pyruvate. Cells were maintained at 5% CO_2_ and 37°C. Antibodies used in this study were rabbit anti-cdGAP (Cell Signaling Technology, Beverly, MA), mouse anti-α-actinin and rabbit anti-fibronectin (Sigma, St. Louis, MO), mouse anti-paxillin clone 349, and mouse anti-ILK (BD Bioscience, Bedford, MA), and rabbit anti-paxillin clone H114 (Santa Cruz Biotechnology, Santa Cruz, CA).

U2OS cells were transfected with a non-specific control siRNA or two separate siRNAs to human cdGAP. U2OS cells were transfected using Oligofectamine (Invitrogen/Life Technologies, Carlsbad, CA) as per the manufacturer's instructions with siRNAs used at a final concentration of 0.3 μM. The siRNA sequences directed against human cdGAP were as follows (Ambion, Grand Island, NY): cdGAP(1) 5′-GGACAGAUCUCUACAUAGA-3′, cdGAP(2) 5′-CCUCAGCGGAGAUCAGUAA-3′. The control siRNA sequence was 5′ACUCUAUCUGCACGCUGAC-3′. Transfection of tagged proteins into U2OS cells to generate stable vinculin-YFP and zyxin-GFP expressing cell lines was performed with Lipofectamine LTX (Invitrogen/Life Technologies) according to the manufacturer's instructions. The Raichu Rac1 FRET probe was obtained from Dr. Michiyuki Matsuda. U2OS cells were initially selected with 1 mg/ml G418 to produce cell populations with heterogenous expression, and then maintained in 300 μg/ml G418 during culture with the exception of siRNA treatments, where cells were cultured in antibiotic free media.

### Generation of PDMS Substrates

Sylgard 184 (Dow Corning, NY) was thoroughly mixed at either 90∶1 (w/w) or 10∶1 (w/w) to create soft and hard substrates with a Young's modulus of ∼1 kPa and ∼1 MPa, respectively. PDMS mixtures were spun down in a centrifuge for 5 minutes at 500×g to remove any air bubbles introduced by mixing. For experiments on glass coverslips, 50 μl of PDMS was pipetted and any newly formed air bubbles were removed with a needle. For MatTek dishes, 15 μl PDMS was placed onto the center of a MatTek dish, and the dish was placed onto a home-made spin coater. Dishes were spun at ∼3,000 RPM for 15 seconds to spread the PDMS into a thin, even coating on the glass insert in the middle of the dish. After PDMS had been applied, coverslips or dishes were cured in an oven at 70°C overnight. PDMS substrates were sterilized with UV irradiation for 10 minutes, coated with 10 μg/ml fibronectin (BD bioscience), and blocked with 1% (w/v) heat denatured Bovine Serum Albumin (Sigma) before use. Equivalent fibronectin coating for soft and hard PDMS substrates, as well as glass coverslips was verified by staining coated slips with an antibody against fibronectin (Sigma). The Shear modulus of PDMS substrates was determined by curing PDMS mixtures at 70°C between the 40 mm parallel plates of a TA Instruments ARG2 Rheometer (New Castle, DE) with small angle oscillatory shear at 1 Hz. Values for the Shear modulus were converted to the reported Young's modulus values using the equation E = 2G(1+v), where E =  Young's modulus, G =  Shear modulus, v =  Poisson's ratio (assuming a Poisson ratio of 0.5 for PDMS).

### Durotaxis Assay

For durotaxis experiments, 6-well cell culture plates were first filled with 1.0 ml of soft 90∶1 PDMS. After allowing the PDMS to spread for 20 minutes, glass coverslips were carefully placed on top of the PDMS. The edges of the coverslip became overlapped by the uncured PDMS forming a rigidity interface between the PDMS and glass. Plates were cured overnight at 70°C before being sterilized and coated with fibronectin as described above. U2OS cells were plated into chambers for four hours before being imaged in serum-containing DMEM for sixteen hours under phase contrast on a Nikon Eclipse Ti scope under a 10X/0.30 PL FLUOR Nikon objective in an environment chamber at 37°C with regulated CO2. For quantification, a crossing event was considered to have occurred if a cell's nucleus passed over the boundary between the soft PDMS and hard glass, or vice versa. The total number of cells that crossed the boundary, along with the number of times that they crossed, and whether they finished the migration time-lapse analysis on either the soft or hard substrate, was quantified and analyzed using ImageJ and Microsoft Excel.

### Immunofluoresence Microscopy

Cells on fibronectin-coated PDMS were fixed and permeabilized simultaneously using a mixture of 4% (w/v) paraformaldehyde (pH adjusted to 7.2) and 1% (v/v) Triton X-100 in phosphate buffered saline (PBS). Coverslips were washed in PBS containing 0.1% (v/v) Tween 20 (for all wash steps). Fixed coverslips were quenched in PBS containing 0.1 M glycine, before being washed in PBS-Tween 20 and blocked in PBS containing 3% (w/v) BSA. Glass coverslips were incubated with primary antibodies for one hour at room temperature and following three washes in PBS-Tween 20, were incubated for one hour with Dylight-conjugated secondary antibodies (Fisher Scientific, Waltham, MA) in PBS with 3% (w/v) BSA. Filamentous actin was visualized using Rhodamine-phalloidin (Invitrogen, Carlsbad, CA).

### Random Migration Analysis

U2OS cells were plated on spin-coated glass coverslips in 24 well plates for four hours before being imaged in serum containing DMEM for 16 hours under phase contrast on a Nikon Eclipse Ti scope under a 10X/0.30 PL FLUOR Nikon objective in an environment chamber with controlled CO_2_ at 37°C. Velocity and directionality values were obtained by tracking the cell centroid using the Manual Tracking and Chemotaxis Ibidi (Martinsried, Germany) plugins in ImageJ.

### Cell Shape, Adhesion Size, and Cell Area Analysis

Adhesion size was measured in fixed cells using ImageJ from background-subtracted images of paxillin-stained adhesions. Thresholded particles were measured using the Analyze Particles function in ImageJ to give an average adhesion area. For cell area and aspect ratio calculations, thresholded images of the actin channel were analyzed in ImageJ. U2OS cell morphology was quantified using an aspect ratio of the major:minor axis. The major axis in crescent shaped cells ran from one tip of the crescent to the other, and there was a short minor axis from the leading edge of the crescent to the cell rear. In rounded cells, this ratio approached one, given that the cells were closer to circles than crescents.

### Focal Adhesion Dynamics Analysis

For adhesion dynamics analysis, vinculin-YFP or zyxin-GFP expressing cells were transfected with a control siRNA or siRNAs targeting cdGAP. Cells were re-plated onto soft or hard PDMS-coated MatTek glass bottom dishes (MatTek Corp, Ashland, MA) overnight before being imaged on a Leica SP5 confocal microscope using an HCX PL APO 63×/1.40–0.60 OIL λ BL objective (Leica, Bannockburn, IL) equipped with an environment chamber maintained at 37°C with regulated CO_2_. Time-lapse movies were compiled and background subtracted before being analyzed in ImageJ. Only individual adhesions that could be followed from the point at which they were initially visible all the way through until complete disassembly were quantified for lifetime analysis.

### Membrane Dynamics Analysis

For the membrane dynamics analysis, high magnification confocal movies of cells transfected with tagged FA markers were analyzed using the QuimP11 plugin for ImageJ. This plugin calculates the velocity of the entire cell membrane from frame to frame, including both extension and retraction events. The average membrane velocity was calculated by taking the absolute value of all the data points reported by the plugin, as retraction rates were reported as negative values, and then averaging them together to give the overall membrane velocity reported in Figure 1G. For more detail on the specific calculations performed by the plugin, please refer to the following publications [68]–[71].

### Raichu FRET Analysis

U2OS cells were co-transfected with control or cdGAP siRNA, and one day later transfected with a Raichu-Rac1 FRET probe using Lipofectamine LTX. Cells were allowed to recover for 16–20 hours post transfection in antibiotic-free complete media and were plated onto MatTek dishes that were spin coated with a thin layer of soft or hard PDMS (see Production of PDMS substrates). Live cell imaging was performed in a CO_2_ and temperature controlled environment on a Leica AF6000 microscope with 100× Fluotar Apo X objective. CFP and YFP excitation and emission was performed using external Leica fast filter wheels in line with a Leica mercury light source and Cascade Roper (Photometrics) camera, respectively. FRET images were acquired sequentially in the following channels, 1) CFP image with CFP excitation and emission, 2) CFP excitation and YFP emission, 3) YFP excitation and YFP emission. Gain and exposure settings were equivalent for all FRET images taken. FRET Channels 1 and 2 were background subtracted in ImageJ and the ratio of YFP (channel 2)/CFP (channel 1) used to produce the FRET image. Images were pseudo-colored with the rainbow RGB on equivalent scales in ImageJ. For gradient calculations, 20 μm wide line profiles were drawn on 32-bit FRET ratio images from the leading edge to the cell nucleus. To normalize FRET gradients in different sized and shaped cells, an average FRET value was calculated along the line profile for each of twelve distance categories, starting at the nucleus.

### Statistics

All data sets were analyzed with GraphPad PRISM software using student's t-test and are representative of the summed data from at least three independently performed trials, and significance indicated with asterisks (* p<0.05, ** p<0.005, *** p<0.0005). All error bars represent 95% confidence intervals. Results were considered significant when the p value was <0.05.

## Supporting Information

Figure S1 (**A**) Average cell area in control and cdGAP siRNA-treated cells spread on soft and hard PDMS coated coverslips. (**B**) The number of cells plated on either side of the rigidity boundary was equivalent for both the control and cdGAP siRNA treatments in the durotaxis assays.
(TIF)

Movie S1 U2OS cells treated with control siRNA migrating on soft PDMS. Phase contrast images acquired every ten minutes, with movies playing back at ten frames per second.
(AVI)

Movie S2 U2OS cells treated with control siRNA migrating on hard PDMS. Phase contrast images acquired every ten minutes, with movies playing back at ten frames per second.
(AVI)

Movie S3 CdGAP siRNA (2) treated cells migrating on soft PDMS. Phase contrast images acquired every ten minutes, with movies playing back at ten frames per second.
(AVI)

Movie S4 CdGAP siRNA (2) treated cells migrating on hard PDMS. Phase contrast images acquired every ten minutes, with movies playing back at ten frames per second.
(AVI)

Movie S5 U2OS cells stably expressing vinculin-YFP treated with control siRNA migrating on soft PDMS. Fluorescence images acquired every two minutes, with movies playing back at ten frames per second.
(AVI)

Movie S6 U2OS cells stably expressing vinculin-YFP treated with control siRNA migrating on hard PDMS. Fluorescence images acquired every two minutes, with movies playing back at ten frames per second.
(AVI)

Movie S7 U2OS cells stably expressing vinculin-YFP treated with cdGAP siRNA migrating on soft PDMS. Fluorescence images acquired every two minutes, with movies playing back at ten frames per second.
(AVI)

Movie S8 U2OS cells stably expressing vinculin-YFP treated with cdGAP siRNA migrating on hard PDMS. Fluorescence images acquired every two minutes, with movies playing back at ten frames per second.
(AVI)

Movie S9 Phase contrast time-lapse of control siRNA-treated U2OS cells plated in durotaxis chambers. The boundary between soft and hard matrix is marked in frame one. Phase contrast images acquired every ten minutes, with movies playing back at ten frames per second.
(AVI)

Movie S10 Phase contrast time-lapse of cdGAP siRNA-treated U2OS cells plated in durotaxis chambers. The boundary between soft and hard matrix is marked in frame one. Phase contrast images acquired every ten minutes, with movies playing back at ten frames per second.
(AVI)
