# Supplementary figures and images for: The Focal Adhesion-Localized CdGAP Regulates Matrix Rigidity Sensing and Durotaxis
Source: PLoS One. 2014 Mar 14;9(3):e91815. doi: 10.1371/journal.pone.0091815 (PMC3954768; doi:10.1371/journal.pone.0091815)

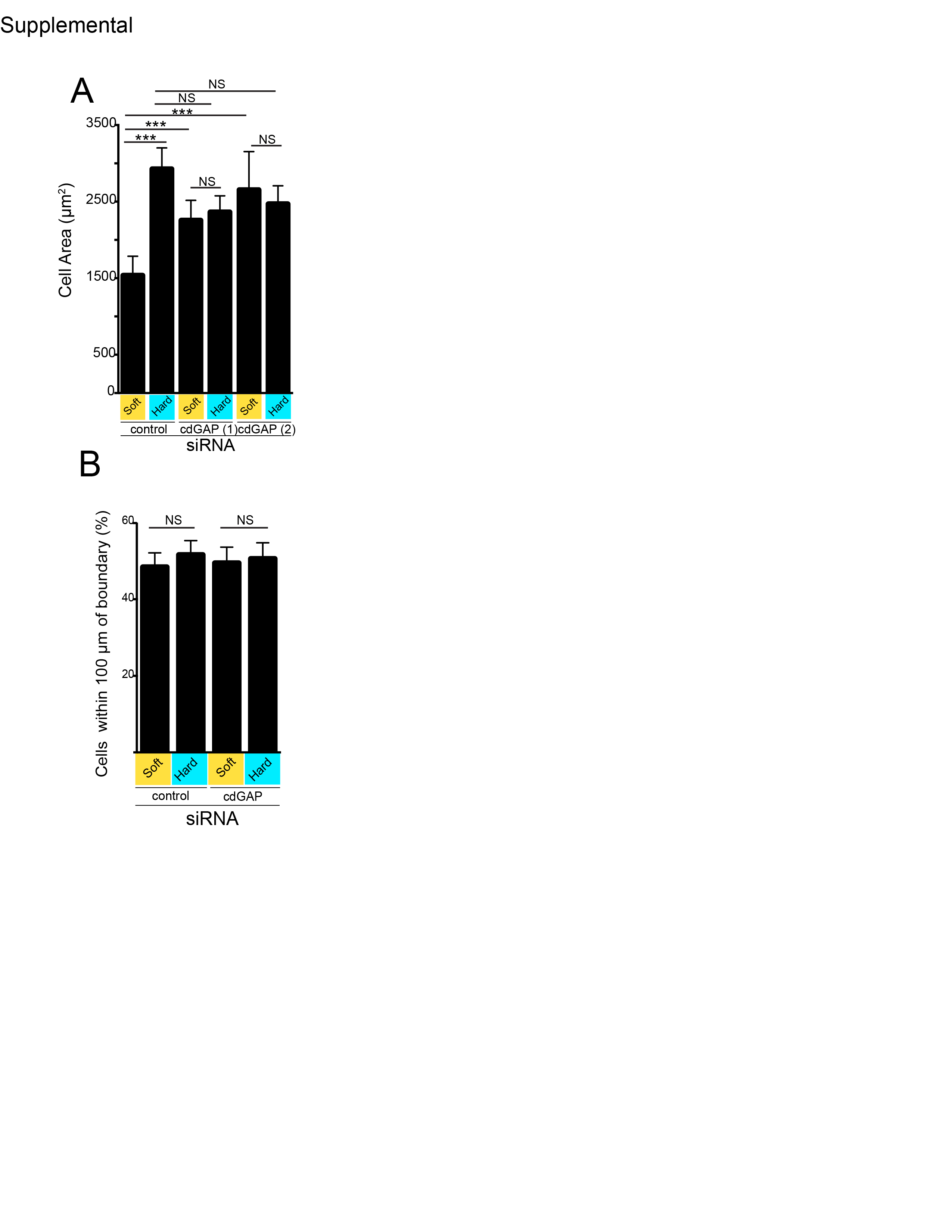

Supplement: Figure S1 — (A) Average cell area in control and cdGAP siRNA-treated cells spread on soft and hard PDMS coated coverslips. (B) The number of cells plated on either side of the rigidity boundary was equivalent for both the control and cdGAP siRNA treatments in the durotaxis assays. (TIF) [file pone.0091815.s001.tif]
